# Supplementary material for: Neuroprognostication value of serum neurofilament light chain for out-of-hospital cardiac arrest: A systematic review and meta-analysis
Source: PLoS One. 2023 Sep 15;18(9):e0290619. doi: 10.1371/journal.pone.0290619 (PMC10503738; doi:10.1371/journal.pone.0290619)
Supplement: S1 File — (DOCX) [file pone.0290619.s002.docx]

Table S1: baseline patient characteristics and the assay methods about detect NfL concentration

| Study | outcome | N | Age | Female | Bystander performed CPR | Time until ROSC (min) | No-flow durationd (SD)(min) | Low-flow durationd (SD)(min) | TTM | Epinephrine administered (SD)(mg) | The NfL's method of measurement |
| --- | --- | --- | --- | --- | --- | --- | --- | --- | --- | --- | --- |
| Hunziker, Sabina 2021 | good | 66(40%) | 60(17) | 11(17%) | 51 (77%) | 16.53(14.30) | 1.7(2.96) | 16.53(14.30) | 40(61%) | 1.08(1.76) | Simoa |
|  | poor | 98(60%) | 65 (14) | 36(37%) | 52 (53%) | 23.12(15.17) | 6.02(6.38) | 23.12(15.17) | 69(70%) | 2.96(2.73) | Simoa |
| Moseby-Knappe, M 2019 | total | 717 | 65.5(13.1) | 137(19.1%) | 511 (71.3%) | 30.8(20.9) |  |  | all |  | Simoa |
| Pouplet, C 2022 | total | 49 | 62.6 (18.7) | 9(18.4%) | 41 (83.7%) |  | 0(0-3.0) | 24.15(18.2) | all | 2.64(1.43) | the EllaTM microfluidic platform |
| Raphael Wurm 2021 | good | 21(30%) | 56.7(18.2) | 5(23.8%) | 19 (90%) | 27.6(12.9) |  |  | all | 3.79(5.02) | Simoa |
|  | poor | 49(70%) | 61.0(16.4) | 12(24.5%) | 37 (71%) | 31.3(25.2) |  |  | all | 4.17(1.94) | Simoa |
| Wihersaari, L. 2021 | good | 73(65.2%) | 59.0(11.4) | 16% | 66 (90.2%) | 18.5(5.4) |  |  | all |  | Simoa |
|  | poor | 39(34.8%) | 67.0(12.1) | 69% | 27 (69.2%) | 26.9(7.2) |  |  | all |  | Simoa |
| Wihersaari, L. 2022 | good | 128(51.6%) |  | 21 (11.8%) | 21 (11.8%) | 18.3(10.7) |  |  | 100(78.1%) |  | Simoa |
|  | poor | 120(49.4%) |  | 19 (15.8%) | 19 (15.8%) | 25.7(9.5) |  |  | 92(76.7%) |  | Simoa |

N: number;

Table S2: Characteristics of include study

**Hunziker, Sabina 2021**

**Patient Selection**

| 1. **Risk of Bias** |  |
| --- | --- |
| Patient Sampling |  |
| Was a consecutive or random sample of patients enrolled? | Yes |
| Was a case-control design avoided? | Yes |
| Did the study avoid inappropriate exclusions? | Yes |
| **Could the selection of patients have introduced bias?** | Low risk |

| **B. Concerns regarding applicability** |  |
| --- | --- |
| Patient characteristics and setting |  |
| **Are there selection that the included patients and setting do not match the review question** | Low concern |

**Index Test**

| Index test |  |
| --- | --- |

**All Tests**

| 1. **Risk of Bias** |  |
| --- | --- |
| Were the test results interpreted without knowledge of the results of the reference standard? | Unclear |
| If a threshold was used, was it pre-specified? | Yes |
| **Could the conduct or interpretation of the index test have introduced bias?** | Unclear risk |

| 1. **Concerns regarding applicability** |  |
| --- | --- |
| **Are there concerns that the index test, its conduct,or interpretation differ from the review question?** | Low concern |

**Reference Standard**

| 1. **Risk of Bias** |  |
| --- | --- |
| Target condition and reference standard(s) |  |
| Is the reference standards likely to correctly classify the target condition? | Yes |
| Were the reference standard results interpreted without knowledge of the results of the index tests? | Yes |
| **Could the reference standard, its conduct, or its interpretation have introduced bias?** | Low risk |

| **B. Concerns regarding applicability** |  |
| --- | --- |
| **Are there concerns that the target condition as defined by the reference standard does not match the question?** | Low concern |

**Flow and Timing**

| 1. **Risk of Bias** |  |
| --- | --- |
| Flow and timing |  |
| Was there an appropriate interval between index test and reference standard? | Unclear |
| Did all patients receive the same reference standard? | Yes |
| Were all patients included in the analysis? | Yes |
| **Could the patient flow have introduced bias?** | Low risk |

**Notes**

| **Notes** |  |
| --- | --- |

**Moseby-Knappe, M 2019**

**Patient Selection**

| 1. **Risk of Bias** |  |
| --- | --- |
| Patient Sampling |  |
| Was a consecutive or random sample of patients enrolled? | Yes |
| Was a case-control design avoided? | Yes |
| Did the study avoid inappropriate exclusions? | Yes |
| **Could the selection of patients have introduced bias?** | Low risk |

| **B. Concerns regarding applicability** |  |
| --- | --- |
| Patient characteristics and setting |  |
| **Are there selection that the included patients and setting do not match the review question** | Low concern |

**Index Test**

| Index test |  |
| --- | --- |

**All Tests**

| 1. **Risk of Bias** |  |
| --- | --- |
| Were the test results interpreted without knowledge of the results of the reference standard? | Unclear |
| If a threshold was used, was it pre-specified? | No |
| **Could the conduct or interpretation of the index test have introduced bias?** | Unclear risk |

| 1. **Concerns regarding applicability** |  |
| --- | --- |
| **Are there concerns that the index test, its conduct,or interpretation differ from the review question?** | Low concern |

**Reference Standard**

| 1. **Risk of Bias** |  |
| --- | --- |
| Target condition and reference standard(s) |  |
| Is the reference standards likely to correctly classify the target condition? | Yes |
| Were the reference standard results interpreted without knowledge of the results of the index tests? | Yes |
| **Could the reference standard, its conduct, or its interpretation have introduced bias?** | Low risk |

| **B. Concerns regarding applicability** |  |
| --- | --- |
| **Are there concerns that the target condition as defined by the reference standard does not match the question?** | Low concern |

**Flow and Timing**

| 1. **Risk of Bias** |  |
| --- | --- |
| Flow and timing |  |
| Was there an appropriate interval between index test and reference standard? | Unclear |
| Did all patients receive the same reference standard? | Yes |
| Were all patients included in the analysis? | Yes |
| **Could the patient flow have introduced bias?** | Low risk |

**Notes**

| **Notes** |  |
| --- | --- |

**Pouplet, C 2022**

**Patient Selection**

| 1. **Risk of Bias** |  |
| --- | --- |
| Patient Sampling |  |
| Was a consecutive or random sample of patients enrolled? | Yes |
| Was a case-control design avoided? | Yes |
| Did the study avoid inappropriate exclusions? | Yes |
| **Could the selection of patients have introduced bias?** | Low risk |

| **B. Concerns regarding applicability** |  |
| --- | --- |
| Patient characteristics and setting |  |
| **Are there selection that the included patients and setting do not match the review question** | Low concern |

**Index Test**

| Index test |  |
| --- | --- |

**All Tests**

| 1. **Risk of bias** |  |
| --- | --- |
| Were the test results interpreted without knowledge of the results of the reference standard? | Unclear |
| If a threshold was used, was it pre-specified? | No |
| **Could the conduct or interpretation of the index test have introduced bias?** | High risk |

| 1. **Concerns regarding applicability** |  |
| --- | --- |
| **Are there concerns that the index test, its conduct,or interpretation differ from the review question?** | Low concern |

**Reference Standard**

| 1. **Risk of bias** |  |
| --- | --- |
| Target condition and reference standard(s) |  |
| Is the reference standards likely to correctly classify the target condition? | Yes |
| Were the reference standard results interpreted without knowledge of the results of the index tests? | Yes |
| **Could the reference standard, its conduct, or its interpretation have introduced bias?** | Low risk |

| **B. Concerns regarding applicability** |  |
| --- | --- |
| **Are there concerns that the target condition as defined by the reference standard does not match the question?** | Low concern |

**Flow and Timing**

| 1. **Risk of Bias** |  |
| --- | --- |
| Flow and timing |  |
| Was there an appropriate interval between index test and reference standard? | Unclear |
| Did all patients receive the same reference standard? | Yes |
| Were all patients included in the analysis? | Yes |
| **Could the patient flow have introduced bias?** | Low risk |

**Notes**

| **Notes** |  |
| --- | --- |

**Raphael Wurm 2021**

**Patient Selection**

| 1. **Risk of Bias** |  |
| --- | --- |
| Patient sampling |  |
| Was a consecutive or random sample of patients enrolled? | Yes |
| Was a case-control design avoided? | Yes |
| Did the study avoid inappropriate exclusions? | Yes |
| **Could the selection of patients have introduced bias?** | Low risk |

| **B. Concerns regarding applicability** |  |
| --- | --- |
| Patient characteristics and setting |  |
| **Are there selection that the included patients and setting do not match the review question** | Low concern |

**Index Test**

| Index test |  |
| --- | --- |

**All Tests**

| 1. **Risk of bias** |  |
| --- | --- |
| Were the test results interpreted without knowledge of the results of the reference standard? | No |
| If a threshold was used, was it pre-specified? | No |
| **Could the conduct or interpretation of the index test have introduced bias?** | High risk |

| 1. **Regarding applicability** |  |
| --- | --- |
| **Are there concerns that the index test, its conduct,or interpretation differ from the review question?** | Low concern |

**Reference Standard**

| 1. **Risk of Bias** |  |
| --- | --- |
| Target condition and reference standard(s) |  |
| Is the reference standards likely to correctly classify the target condition? | Yes |
| Were the reference standard results interpreted without knowledge of the results of the index tests? | Unclear |
| **Could the reference standard, its conduct, or its interpretation have introduced bias?** | Unclear risk |

| **B. Concerns regarding applicability** |  |
| --- | --- |
| **Are there concerns that the target condition as defined by the reference standard does not match the question?** | Low concern |

**Flow and Timing**

| 1. **Risk of Bias** |  |
| --- | --- |
| Flow and timing |  |
| Was there an appropriate interval between index test and reference standard? | Unclear |
| Did all patients receive the same reference standard? | Yes |
| Were all patients included in the analysis? | Yes |
| **Could the patient flow have introduced bias?** | Low risk |

**Notes**

| **Notes** |  |
| --- | --- |

**Wihersaari, L. 2021**

**Patient Selection**

| 1. **Risk of Bias** |  |
| --- | --- |
| Patient Sampling |  |
| Was a consecutive or random sample of patients enrolled? | Yes |
| Was a case-control design avoided? | Yes |
| Did the study avoid inappropriate exclusions? | Yes |
| **Could the selection of patients have introduced bias?** | Low risk |

| **B. Concerns regarding applicability** |  |
| --- | --- |
| Patient characteristics and setting |  |
| **Are there selection that the included patients and setting do not match the review question** | Low concern |

**Index Test**

| Index Test |  |
| --- | --- |

**All Tests**

| 1. **Risk of Bias** |  |
| --- | --- |
| Were the test results interpreted without knowledge of the results of the reference standard? | Yes |
| If a threshold was used, was it pre-specified? | No |
| **Could the conduct or interpretation of the index test have introduced bias?** | Unclear risk |

| 1. **Concerns regarding applicability** |  |
| --- | --- |
| **Are there concerns that the index test, its conduct,or interpretation differ from the review question?** | Low concern |

**Reference Standard**

| 1. **Risk of Bias** |  |
| --- | --- |
| Target condition and reference standard(s) |  |
| Is the reference standards likely to correctly classify the target condition? | Yes |
| Were the reference standard results interpreted without knowledge of the results of the index tests? | Yes |
| **Could the reference standard, its conduct, or its interpretation have introduced bias?** | Low risk |

| **B. Concerns regarding applicability** |  |
| --- | --- |
| **Are there concerns that the target condition as defined by the reference standard does not match the question?** | Low concern |

**Flow and Timing**

| 1. **Risk of Bias** |  |
| --- | --- |
| Flow and timing |  |
| Was there an appropriate interval between index test and reference standard? | Yes |
| Did all patients receive the same reference standard? | Yes |
| Were all patients included in the analysis? | Yes |
| **Could the patient flow have introduced bias?** | Low risk |

**Notes**

| **Notes** |  |
| --- | --- |

**Wihersaari, L. 2022**

**Patient Selection**

| 1. **Risk of Bias** |  |
| --- | --- |
| Patient sampling |  |
| Was a consecutive or random sample of patients enrolled? | unclear |
| Was a case-control design avoided? | Yes |
| Did the study avoid inappropriate exclusions? | Yes |
| **Could the selection of patients have introduced bias?** | Low risk |

| **B. Concerns regarding applicability** |  |
| --- | --- |
| Patient characteristics and setting |  |
| **Are there selection that the included patients and setting do not match the review question** | Low concern |

**Index Test**

| Index test |  |
| --- | --- |

**All Tests**

| 1. **Risk of Bias** |  |
| --- | --- |
| Were the test results interpreted without knowledge of the results of the reference standard? | Unclear |
| If a threshold was used, was it pre-specified? | No |
| **Could the conduct or interpretation of the index test have introduced bias?** | High risk |

| 1. **Concerns regarding applicability** |  |
| --- | --- |
| **Are there concerns that the index test, its conduct,or interpretation differ from the review question?** | Low concern |

**Reference Standard**

| 1. **Risk of Bias** |  |
| --- | --- |
| Target condition and reference standard(s) |  |
| Is the reference standards likely to correctly classify the target condition? | Yes |
| Were the reference standard results interpreted without knowledge of the results of the index tests? | Unclear |
| **Could the reference standard, its conduct, or its interpretation have introduced bias?** | Unclear risk |

| **B. Concerns regarding applicability** |  |
| --- | --- |
| **Are there concerns that the target condition as defined by the reference standard does not match the question?** | Low concern |

**Flow and Timing**

| **A.Risk of Bias** |  |
| --- | --- |
| Flow and timing |  |
| Was there an appropriate interval between index test and reference standard? | Unclear |
| Did all patients receive the same reference standard? | Yes |
| Were all patients included in the analysis? | Yes |
| **Could the patient flow have introduced bias?** | Low risk |

**Notes**

| **Notes** |  |
| --- | --- |


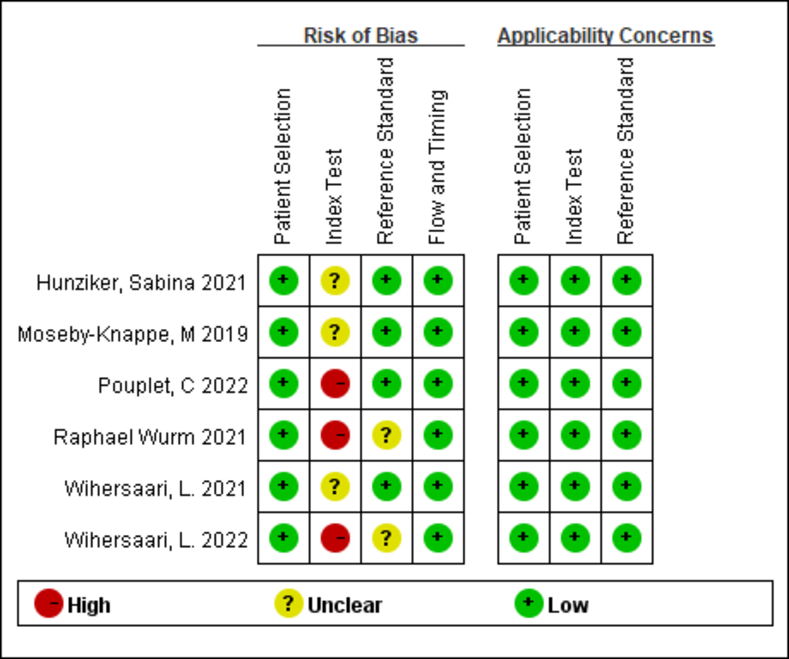


S1a Fig : Summary of assessment of the risk of bias in the included studies


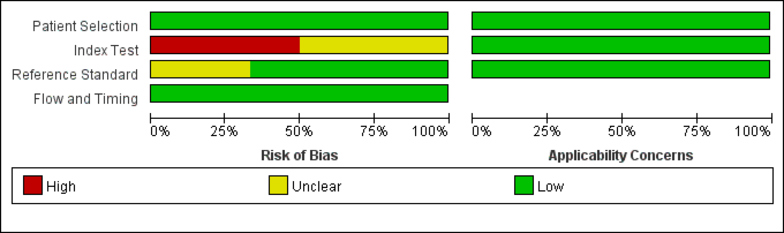


S1b Fig : Summary of assessment of the risk of bias in the included studies


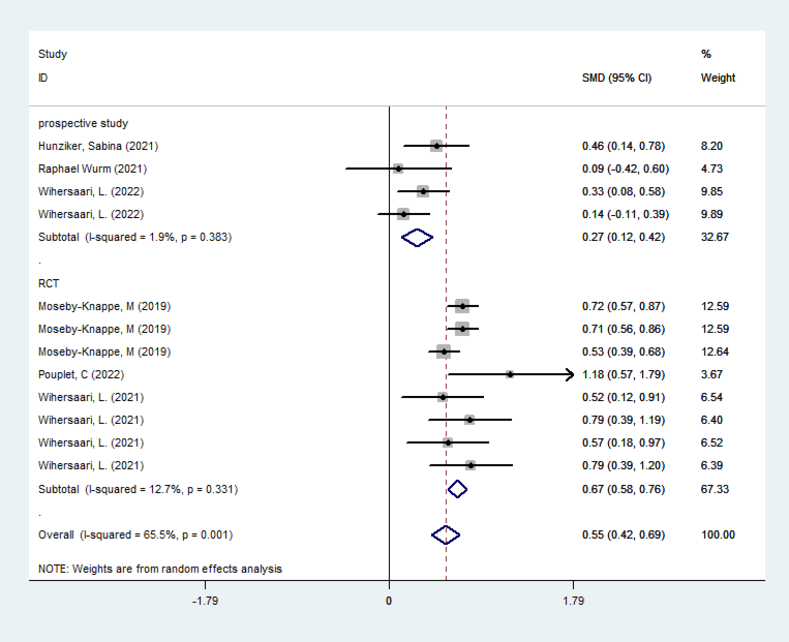


S2 Fig : Analysis by study type as subgroup

Abbreviations: CI, confidence interval; SD, standard deviation


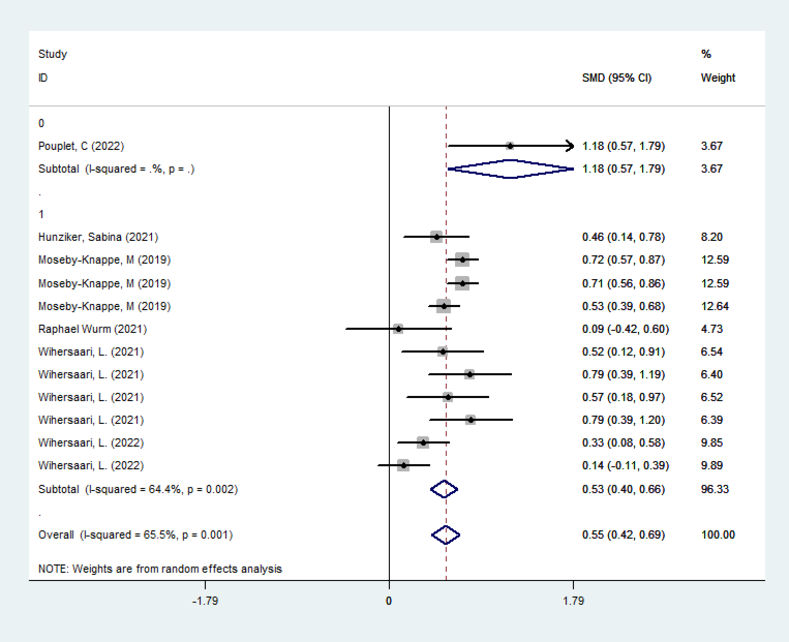


S3 Fig: Analysis by instrument to measure the NfL as subgroup

0: using the Ella^TM^ microfluidic platform;

1: using the simoa.

Abbreviations: CI, confidence interval; SD, standard deviation


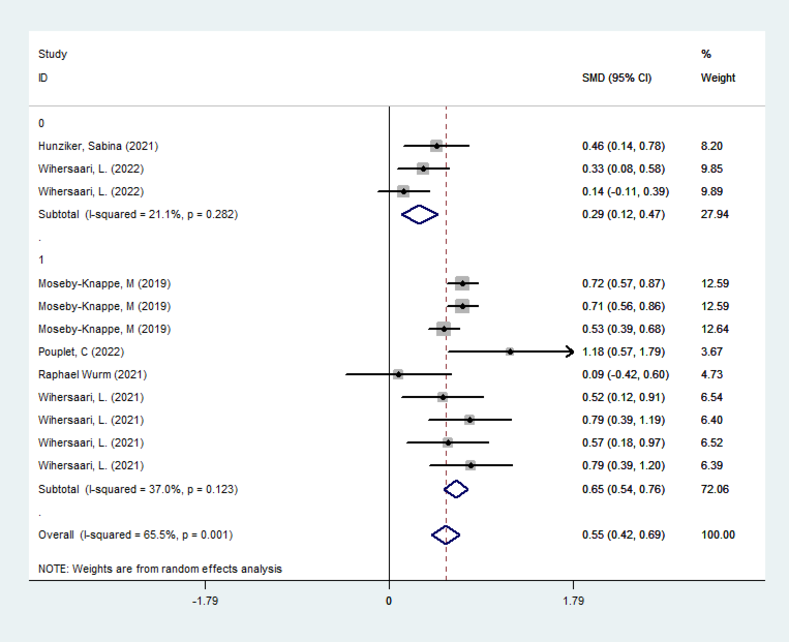


S4 Fig :Analysis according to receive TTM treatment

0: Only some of the patients in the study received TTM;

1: All patients in the study were treated with TTM.

Abbreviations: CI, confidence interval; SD, standard deviation


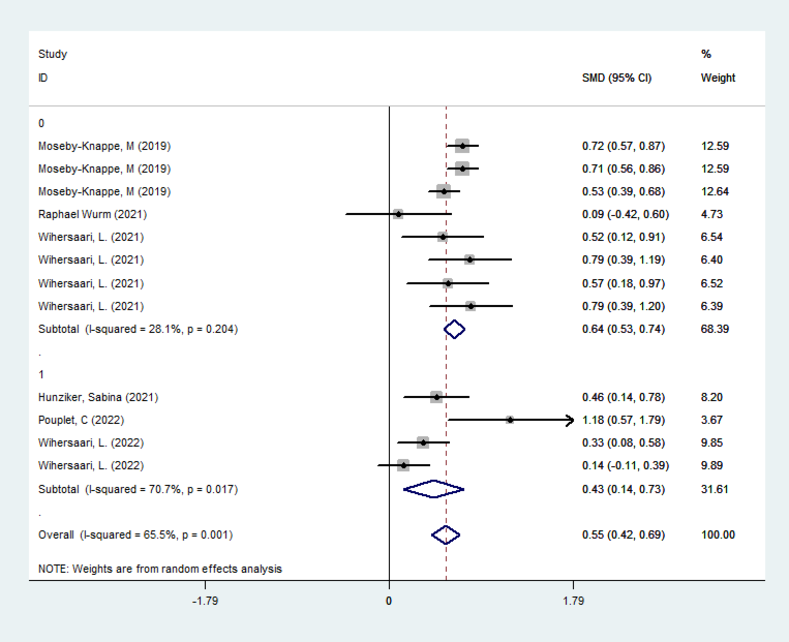


S5 Fig: Subgroup analysis according to subjects with or without of neurological disease.

0: The patient had no neurological disease;

1: The patient had a previous neurological condition or cardiac arrest with a neurological condition as the cause.

Abbreviations: CI, confidence interval; SD, standard deviation


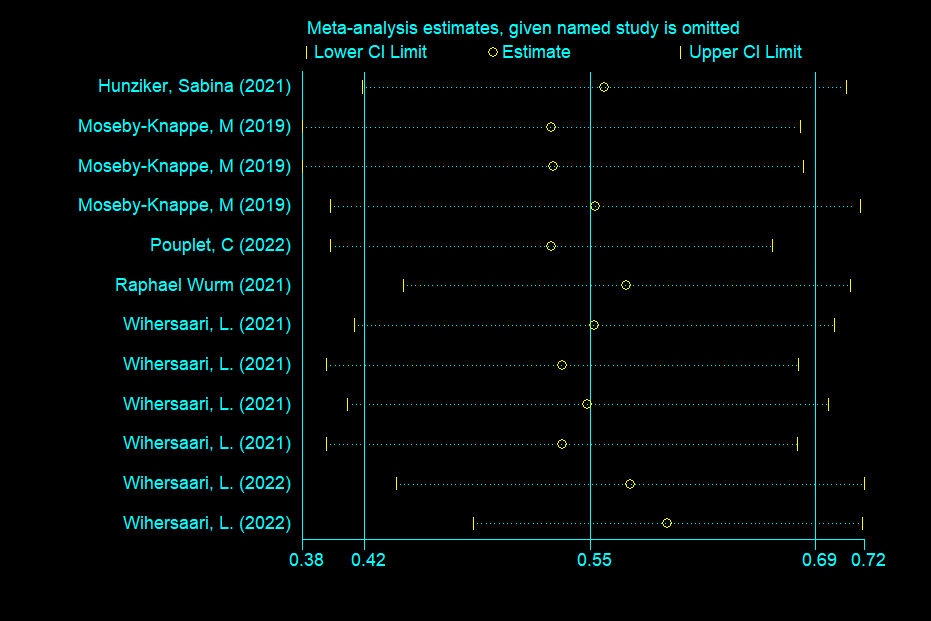


S6 Fig: The sensitivity analysis of the included studies

Abbreviations: CI, confidence interval


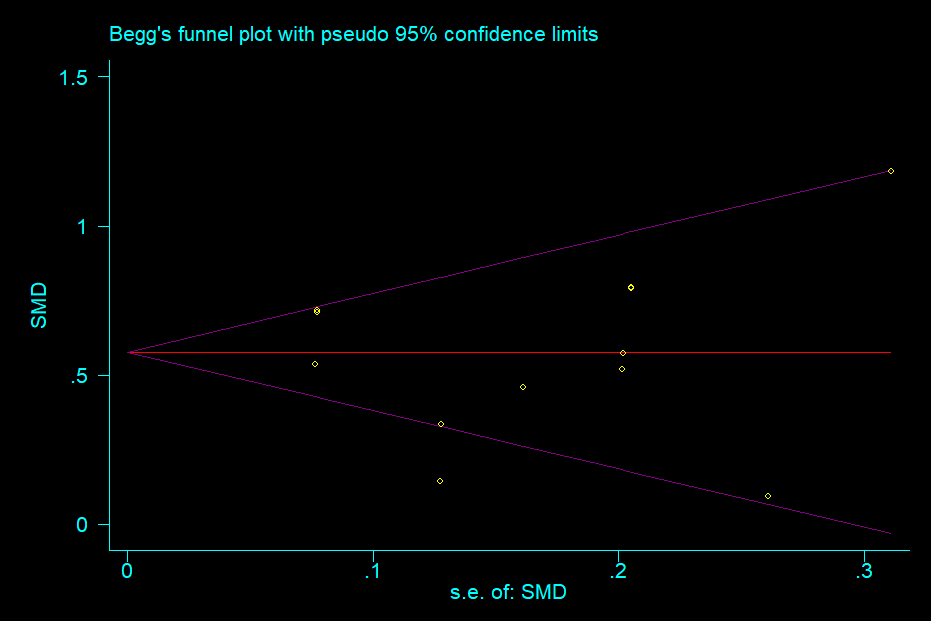


S7a Fig : the publication bias of Begg's funnel plot





S7b Fig : the publication of Begg’s test and Egger’s test
