# Supplementary material for: Neuroprognostication value of serum neurofilament light chain for out-of-hospital cardiac arrest: A systematic review and meta-analysis
Source: PLoS One. 2023 Sep 15;18(9):e0290619. doi: 10.1371/journal.pone.0290619 (PMC10503738; doi:10.1371/journal.pone.0290619)
Supplement: S2 File — (DOCX) [file pone.0290619.s003.docx]

|  |  |  | 24hour | | | 48hour | | | 72hour | | | on admission | | |
| --- | --- | --- | --- | --- | --- | --- | --- | --- | --- | --- | --- | --- | --- | --- |
| Study | Publication year |  | number | NfL M(Q1-Q3) | | number | NfL M(Q1-Q3) | | number | NfL M(Q1-Q3) | | number | NfL M(Q1-Q3) | |
| Raphael Wurm | 2021 | GNO |  |  |  | 21 | 18.5 | 11.3-28.2 |  |  |  | 21 | 16.5 | 9.35-21.4 |
|  |  | PNO |  |  |  | 49 | 297 | 39.7-1572 |  |  |  | 49 | 641 | 197-2173 |
| Moseby-Knappe, M | 2019 | GNO | 351 | 37 | 20-70 | 351 | 46 | 26-101 | 351 | 54 | 30-122 |  |  |  |
|  |  | PNO | 360 | 1426 | 299-3577 | 360 | 3240 | 623-8271 | 360 | 3344 | 845-7838 |  |  |  |
| Wihersaari, L. | 2021 | GNO | 73 | 12.1 | 8.3–23.7 | 73 | 19.1 | 11–30.7 | 73 | 20.5 | 13.8–34.8 | 73 | 10.4 | (7.1–16) |
|  |  | PNO | 39 | 761.9 | 217.6–1534.9 | 39 | 2342.6 | 586.9–5828.8 | 39 | 1727.9 | 643.1–4583.5 | 39 | 13.4 | (10.5—23.2) |
| Pouplet, C | 2022 | GNO |  |  |  | 26 | (mean)72 | (sd) 78 |  |  |  |  |  |  |
|  |  | PNO |  |  |  | 23 | (mean)7,755.50 | (sd) 9,501.1 |  |  |  |  |  |  |
| Wihersaari, L. | 2022 | GNO | 128 | 30.9 | 16.9–61.2 | 128 | 35.6 | 21.3–86.7 |  |  |  |  |  |  |
|  |  | PNO | 120 | 688.9 | 4.1-1803.8 | 120 | 1162.4 | 146.8-4360.5 |  |  |  |  |  |  |
| Hunziker, Sabina | 2021 | GNO | 66 | 27 | 13-46 |  |  |  |  |  |  |  |  |  |
|  |  | PNO | 98 | 116 | 41-330 |  |  |  |  |  |  |  |  |  |
