# Supplementary material for: Neuroprognostication value of serum neurofilament light chain for out-of-hospital cardiac arrest: A systematic review and meta-analysis
Source: PLoS One. 2023 Sep 15;18(9):e0290619. doi: 10.1371/journal.pone.0290619 (PMC10503738; doi:10.1371/journal.pone.0290619)
Supplement: Attachment — Submitted filename: Response to Reviewers.docx [file pone.0290619.s004.docx]

Dear editor:

Thank you for your review of our articles and your valuable comments. Here are our responses to reviewers' comments.

Requirements for journals:

1.We have modified according to the writing requirements of PLOS ONE.

2.Our article was written in accordance with the PRISMA checklist, and its corresponding content is submitted again.

3.We have made changes to our financial disclosures and completed the changes in “cover letter”. Our article was funded by a Science and Technology Project of Hebei Province (approval number :20377764D) in our province.

Funders help us complete the revision of the article and provide financial support.

4.The minimum basic data set of the study has been submitted to the website of this journal as a supporting information file.

5.The title of the supporting information file has been added at the end of the manuscript.

Answers to the following questions:

1.Is the manuscript technically sound, and do the data support the conclusions?

Our article was written in accordance with PRISMA's checklist, and some content may not be submitted in time, which has been added in this revision.

2.Has the statistical analysis been performed appropriately and rigorously?

When collecting data, we found that the available data provided in the included studies were in the form of median and standard deviation, and the original data could not be found, so we adopted a data conversion formula for data processing. Our research has been completed, and this formula has been proved reliable and adopted by many research institutes.(Hoiland et al., 2022, JAMA neurology; Li et al., 2022, The Lancet Respiratory medicine )

3.Have the authors made all data underlying the findings in their manuscript fully available?

The minimum basic data set of the study has been submitted to the website of this journal as a supporting information file.

4.Is the manuscript presented in an intelligible fashion and written in standard English?

The language of our article is English.

Reply to Reviewer #1：

1. Abstract, methods>prognosis analysis:

The standard mean difference and 95% confidence interval were calculated using the random-effects model or fixed-effects model to assess the association between one variable factor NfL level and the outcome of CA patients.

2. Methods (both in abstract and in results)>it should be clearly stated if these results derive from univariate or multivariate analysis

Assess the association between one variable factor NfL level and the outcome of CA patients.

3.Abstract>conclusion.The authors wrote "regardeless on then samples were collected". This was not reported in the results, so they should either report in the results or remove from the conclusion.

"regardeless on then samples were collected" that means 24 hours, 48 hours and 72 hours after cardiac arrest. Statistics for three points in time have been added to the results section.

4.methods>exclusion of patients younger than 16 years should be discussed.

The subjects included in the study are adults. Due to the differences in regional population differentiation, people under the age of 16 are excluded.

5.methhods>conversion of unit of measures into quertiles, means etc was performed throuhg online calculator (please add reference).

Hoiland et al., JAMA neurology 2022; Li et al., The Lancet Respiratory medicine 2022; Su et al., Medicina (Kaunas, Lithuania) 2021.

6.methods>primary end point should be defined.

The results we were interested in were whether nfl could predict the prognosis of patients with cardiac arrest.

7.methods>it is not clear if the correlation derived (for eacht study) from multivariate analysis or not. This is a crucial point.

The data for statistical analysis were all from similar populations, and the data were processed according to time and detection methods.

8.methods>subgroup analuysis for rct vs. non rct should be added.

We have analyzed the ability of study types to predict outcomes in NfL in a subgroup analysis.(Figure S2).

9.discussion; the results are strong, although they should be discussed cautiosuly.

NfL levels were increased in the poor prognosis group compared with the good prognosis group, with high levels of NfL may indicate a poor prognosis in patients with out-of-hospital cardiac arrest.

Reply to Reviewer #2:

1. Although meta-analysis has confirmed its predictive value, it has not conducted a more detailed analysis of its research. We conducted a meta-analysis to evaluate the relationship between serum NfL level and neurological prognosis in patients with spontaneous circulation recovery after CA, and subgroup analysis of the included studies was conducted to evaluate the influence of other factors on the predictive value of NfL.

2.We have filled in the missing parts proposed in the article, and compared the two articles mentioned by the reviewer.

3.The NfL data included in the study were not normally distributed data, but the mean and standard deviation were required for statistical analysis, so we converted the data, and the formula we used was proved to be reliable by other studies and has been used by many studies.( Hoiland et al., 2022, JAMA neurology; Li et al., 2022, The Lancet Respiratory medicine )

4.Exclude the reasons for the following articles：

Adler et al. 2021; PMID: 34328545: It did not use cerebral performance category score (CPC) to evaluate outcomes.

Disanto et al. 2019; PMID: 31375414: Fourteen patients were included in this paper, but 15 cardiac arrests were recorded, and the timing of specimen collection was not stated.

Hoiland e; t al. 2021PMID: 34287000: The study did not provide criteria or timing for assessing nervous system function.

Huesgen et al. 2021; PMID: 34223394: The study included a very different population than other studies.

Moseby-Knappe et al. 2021; PMID: 34417831: This study was incorporated into our analysis

Rana et al. 2013; PMID: 23287695: It did not use cerebral performance category score (CPC) to evaluate outcomes.

5.Figure 3：0: NfL specimens were collected on admission; 1: Samples were collected 24 hours after CA; 2: Specimens were obtained 48 hours after CA; 3: Specimens were obtained 72 hours after CA.

6. We added the NfL roc curve to the article to provide more intuitive evidence

7. At the original line 174, we added the following:

Of the mature myelinated axons, neurofilaments are the most abundant protein. Damage and physiological turnover of neurons in the central nervous system (CNS) result in the release of nerve filaments. As a structural protein, NfL is released into the cerebrospinal fluid and blood when neurons are damaged or apoptotic. As a neuron-specific marker of neurological injury, elevated NfL levels can be found in a variety of conditions involving neuroaxonal injury in both the central and peripheral nervous system. In neurodegenerative diseases, NfL could serve as both a prognostic marker of decline but also an efficacy biomarker of experimental therapies. In a meta-analysis of Alzheimer’s disease, frontotemporal and amyotrophic lateral sclerosis, plasma NfL levels were elevated in patients compared to controls with utility in differentiating neurodegenerative conditions from non-neurodegenerative mimics.

Reply to Reviewer #3:

Abstract:-- Major comments:

We made the following modifications: “Although meta-analysis has confirmed its predictive value, it has not conducted a more detailed analysis of its research. We conducted a meta-analysis to evaluate the relationship between serum NfL level and neurological prognosis in patients with spontaneous circulation recovery after CA, and subgroup analysis of the included studies was conducted to evaluate the influence of other factors on the predictive value of NfL.”

Minor comments:

CPC: cerebral performance category score

SMD: standardized mean difference

CI:confidence interval

The main text:

Introduction--Major comments:

“In recent years, there have been attempts to use light chain protein of neural filament to evaluate the prognosis of patients with cardiac arrest. In addition, a systematic review indicated that the NSE threshold for predicting adverse outcomes varied greatly, which may be related to the non-brain-derived origin of NSE, while the predictive value of NfL was higher than that of other biomarkers. However, only two studies in this meta-analysis measured NfL5. Another meta-analysis also confirmed that NfL had the best predictive value, but this meta-analysis did not conduct subgroup analysis of the included studies and could not prove whether the predictive value of NfL was affected by other factors”

Minor comments:

The three sentences mentioned by the Reviewer #3 have been removed, and the preamble has been substantially altered.

Methods--Major comments:

The abbreviations we use include CA, CPR, ROSC, NfL. But we didn't use out-of-hospital cardiac arrest and ventricular fibrillation.We added the ROC curve to the NfL's predictive power.

Results:

Meta-analysis--Major comments:

In the admission: SMD:0.48,95%CI:0.24-0.73; Samples were collected 24 hours after CA: SMD:0.60,95%CI:0.32-0.88;Specimens were obtained 48 hours after CA: SMD:0.51, 95%CI:0.18-0.85;Specimens were obtained 72 hours after CA: SMD:0.59, 95%CI:0.38-0.81

We were unable to obtain serum NFL values for every patient included in the study, so the ROC curve could not be completed.

Minor comments:

Figure 2 shows the inclusion of results from each study at each point in time

Figure 4: 0: NfL specimens were collected on admission; 1: Samples were collected 24 hours after CA; 2: Specimens were obtained 48 hours after CA; 3: Specimens were obtained 72 hours after CA.

Discussion--Major comments:

The discussion section has been modified and references added.

6. PLOS authors have the option to publish the peer review history of their article (what does this mean?). If published, this will include your full peer review and any attached files.

Yes

Yours sincerely,

Fu Yu
