# Supplementary material for: Neuroprognostication value of serum neurofilament light chain for out-of-hospital cardiac arrest: A systematic review and meta-analysis
Source: PLoS One. 2023 Sep 15;18(9):e0290619. doi: 10.1371/journal.pone.0290619 (PMC10503738; doi:10.1371/journal.pone.0290619)
Supplement: Attachment — Submitted filename: Response to Reviewers.docx [file pone.0290619.s005.docx]

**Dear editor:**

Thank you for your review and decision regarding our manuscript, titled “Neuroprognostication Value of Serum Neurofilament Light Chain for Out-of-hospital Cardiac Arrest: A Systematic Review and Meta-Analysis”. We appreciate the reviewers’ and editor’s insightful and helpful comments on our manuscript.

We sincerely thank all reviewers for their insightful comments. We have provided a point-by-point response to the reviewers’ comments. The changes in the revised manuscript are highlighted.

**Response to the reviewer’s comments**

1. **If the authors have adequately addressed your comments raised in a previous round of review and you feel that this manuscript is now acceptable for publication, you may indicate that here to bypass the “Comments to the Author” section, enter your conflict of interest statement in the “Confidential to Editor” section, and submit your "Accept" recommendation.**

We have amended and recovered the issues raised.

2. **Is the manuscript technically sound, and do the data support the conclusions?**

**Response:** After rigorous selection of articles, statistical analysis was carried out on the data included in the study, and appropriate conclusions were drawn according to the data analysis results.

3. **Has the statistical analysis been performed appropriately and rigorously?\**

Data extraction, selection and application of statistical analysis methods were completed by two researchers independently, and the results were consistent. Moreover, the statistical methods we used were introduced in detail in the methodological section.

4. **Have the authors made all data underlying the findings in their manuscript fully available?**

We put the main data in the article, and the rest of the data is uploaded as supporting information.

5. **Is the manuscript presented in an intelligible fashion and written in standard English?**

The language used in our article is English and has been revised.

6. **Review Comments to the Author**

Our paper is a statistical analysis of the data from the original research, and there is no research ethics issue, and the paper will not be published twice.

**Responses to reviewer #1：**

1) **major issues. In the abstract and in the introduction authors stated that others meta analysis have been planned, although this should be better described. in particular it should be added which new informations will be provived by the present paper**

We appreciate the reviewer’s comments. We agree with the reviewer’s concerns about the purpose of the article is not clear. We added the statement of the research purpose in the abstract and the preface respectively.

In the background of the abstract, we have added "We conducted a meta-analysis to evaluate the relationship between serum NfL level and neurological prognosis in patients with spontaneous circulation recovery after CA, and subgroup analysis was conducted according to sample collection time, time to assess neurological function, study design, whether TTM was received, the method of specimen determination, and the presence of neurological disease in patients. To analyze the influence of these factors on the predictive value of serum NfL." **(Line18-23)**

At the end of the preface, we have added "Herein, we performed a meta-analysis to assess the prognostic value of NfL in cardiac arrest patients. In addition, subgroup analysis was performed on sample collection time, neurological function assessment time, study design, acceptance of TTM, specimen determination method, and the presence of neurological disease in patients to assess the influence of these factors on the predictive value of serum NfL."**(Line65-69)**

2) **definition of good and poor prognosis should be better described in the methods**

The neurological outcomes were classified into good and poor groups based on CPC scores, we defined the group with a good prognosis as patients with a CPC score of 1-2, that is, patients with a good neurological outcome(GNO), and patients with a CPC3-5 score as patients with a poor neurological prognosis(PNO).**(Line104-107)**

3) **In the analysis it is not clear when authors used random effect**

It has been proposed in line 123-125 that when *I^2^*>75%, using the random effects model.

4) **subgroup analysis should be performed for rct vs. Observational**

The study type was statistically analyzed as a subgroup and showed no effect on the predictive value of serum NfL.**(Line181-182)**

5) **meta-regression for age, gender and time of rcp should be performed**

We conducted meta-regression analysis on the age, gender and PCR time of each study population, and found no statistical significance.**(Line195-197)**

**Responses to reviewer #3**

**In lines 204-205 You state: “Traditional biomarkers, such as NSE and S100β, are not affected by drugs or hypothermia and have high sensitivity but poor specificity”. Those markers are more specific than sensitive, and overall, the specificity for each biomarker can be assessed very high (99-100%) BUT when the prognostic accuracy (the ability to discriminate according to outcome) is poor, the sensitivity remains low. This underlines the importance of using methods with high discriminative accuracy (high AUROCs) as NfL is. I suggest rewriting this sentence.**

We rewrote the sentence: “Biomarkers are not affected by drugs or hypothermia. The ideal biomarker should be expressed specifically and exclusively. Conventional biomarkers, such as NSE, showed low sensitivity when their specificity was assessed very high, and remained low sensitivity when their specificity was reduced. This underscores the importance of using methods such as the NfL with high discriminant accuracy.”

**In lines 221-224 You say that admission and 72 h NfL has higher prognostic accuracy than at 24-48 h. This is not in line with our findings and with those of Moseby-Knappe et al. In the COMACARE population, the admission NfL had only moderate accuracy, whereas it was excellent at 24, 48 and 72 h similarly to in the TTM analysis. In a recent analysis, NfL had slightly better (and not inferior) ability at 48 h than at 12 h and admission sample had only satisfactory prognostic ability (Levin H, Lybeck A, Frigyesi A, Arctaedius I, Thorgeirsdóttir B, Annborn M, Moseby-Knappe M, Nielsen N, Cronberg T, Ashton NJ, Zetterberg H, Blennow K, Friberg H, Mattsson-Carlgren N. Plasma neurofilament light is a predictor of neurological outcome 12 h after cardiac arrest. Crit Care. 2023 Feb 24;27(1):74 ). The kinetic of NfL seems to be that it rises 12-24 h after CA and the prognostic accuracy remains high between 24 and 72 h. You analyzed admission NfL only in a one study. Even if Your analysis suggests those results (maybe affected by heterogeneity) I suggest modifying this chapter and do not suggest the superiority of admission NfL because the studies do not support this. Moreover, You should modify the conclusion accordingly.**

We revised the conclusion: “We analyzed predictive levels of NfL at various time points and found that NfL levels at admission and 72 hours after CA were highly prognostic, but only one study has completed the collection of NfL at admission. Therefore, although our results are satisfactory, more studies are needed to confirm one idea. Serum NfL also had good predictive value at 24 and 48 hours, with high heterogeneity, while its predictive value at 24 and 48 hours was equivalent to that at 72 hours among COMACARE populations17. Similarly, a recent study found that the predictive value 48 hours after CA was better than at admission22. Therefore, we have reason to believe that the prediction ability of serum NfL at 24 and 48 hours after CA is better after increasing the sample size.”

**Lines 225-230: I am asking if the outcome definition at 3 mth offers any benefit compared to 6-mth outcome. Most studies define outcome at 6 months. Maybe “the best CPC or mRS” would offer some additional benefit in studying the prognostic accuracy of neuronal biomarkers?**

The neurological function evaluation of cerebrovascular diseases is generally conducted 3 months after the occurrence of cerebrovascular accidents. Therefore, there is a certain basis for evaluating the neurological function prognosis of patients 3 months after CA. Therefore, we believe that we can increase the research of neurological function evaluation 3 months after CA in the future.

We appreciate your re-evaluation of our revised manuscript. We would be glad to respond to any further questions and comments that you may have.

Sincerely,

Zhi-Guo Chen
