# Supplementary material for: Neuroprognostication value of serum neurofilament light chain for out-of-hospital cardiac arrest: A systematic review and meta-analysis
Source: PLoS One. 2023 Sep 15;18(9):e0290619. doi: 10.1371/journal.pone.0290619 (PMC10503738; doi:10.1371/journal.pone.0290619)
Supplement: Attachment — Submitted filename: Response to Reviewers.docx [file pone.0290619.s006.docx]

Dear editor:

Thank you for your review and decision regarding our manuscript, titled “Neuroprognostication Value of Serum Neurofilament Light Chain for Out-of-hospital Cardiac Arrest: A Systematic Review and Meta-Analysis”. We appreciate the reviewers’ and editor’s insightful and helpful comments on our manuscript.

We sincerely thank all reviewers for their insightful comments. We have provided a point-by-point response to the reviewers’ comments. The changes in the revised manuscript are highlighted.

None of the references we cited have been retracted.

**Response to the reviewer’s comments**

**"The neurological function evaluation of cerebrovascular diseases is generally conducted 3 months after the occurrence of cerebrovascular accidents. Therefore, there is a certain basis for evaluating the neurological function prognosis of patients 3 months after CA. Therefore, we believe that we can increase the research of neurological function evaluation 3 months after CA in the future."**

**This is not an acceptable logic. Stroke is a very different entity compared to post-resuscitation. Especially as your own results show that the only available data does not support neuroprognostication at 3 months there is no basis to endorse it here. Moreover, instead of blindly suggesting "more research is needed" on this, as is routine, you could analyze if any are needed (PMID: 37338877).**

In subgroup analyses based on the time point of assessment, serum NfL concentration was found to be predictive of neurological function at discharge, 6 months, and 12 months after CA, but the neuroprognostication value at 3 months is unclear. However, only one study evaluated neurological function at 3 months after CA, in which the level of NFL had no neuroprognostication value at each time point of assessment. Our study may have enrolled more severe patients, creating heterogeneity with other studies (registration website: https://clinicaltrials.gov/ct2/show/NCT02555254). At present, there are few studies on the evaluation of neurological function in CA patients at three months, which is insufficient to support our conclusions.

We appreciate your re-evaluation of our revised manuscript. We would be glad to respond to any further questions and comments that you may have.

Sincerely,

Zhi-Guo Chen
